# *Diplotaxis erucoides* and *Oxalis pes-caprae*: Two Wild Edible Plants as a New and Valuable Source of Carotenoids, Tocols and B1 and B2 Vitamins

**DOI:** 10.3390/nu16142293

**Published:** 2024-07-17

**Authors:** Jesús Clemente-Villalba, Alessandra Fratianni, Hanán Issa-Issa, Giuseppe Ianiri, Francisca Hernández, Caroline Vitone, Ángel A. Carbonell-Barrachina, Gianfranco Panfili

**Affiliations:** 1 Grupo de Investigación Calidad y Seguridad Alimentaria, Instituto de Investigación e Innovación Agroalimentaria y Agroambiental (CIAGRO-UMH), Miguel Hernández University, Ctra. Beniel, km 3.2, 03312 Orihuela, Spain; jesus.clemente@hotmail.com (J.C.-V.); hissa@umh.es (H.I.-I.); 2 Department of Agricultural, Environmental and Food Sciences, University of Molise, Via De Sanctis, 86100 Campobasso, Italy; g.ianiri@studenti.unimol.it (G.I.); c.vitone@studenti.unimol.it (C.V.); panfili@unimol.it (G.P.); 3 Grupo de Investigación en Fruticultura y Técnicas de Producción, Instituto de Investigación e Innovación Agroalimentaria y Agroambiental (CIAGRO-UMH), Miguel Hernández University, Ctra. Beniel, km 3.2, 03312 Orihuela, Spain; francisca.hernandez@umh.es

**Keywords:** WEPs, carotenoids, tocols, riboflavin, thiamine

## Abstract

The aim of this study was to determine the profile and contents of carotenoids, tocols and B1 and B2 vitamins in different parts of two wild edible plants (WEPs), *Diplotaxis erucoides* and *Oxalis pes-caprae*. Results showed interesting amounts of these bioactive compounds in the leaves, with intakes higher than the Recommended Daily Allowance (RDA) for vitamin A and vitamin E after consumption of 100 g. *Diplotaxis erucoides* and *Oxalis pes-caprae* leaves evidenced high amounts of carotenoids, such as lutein (about 8 mg/100 g and 5 mg, respectively) and β-carotene (about 8 mg/100 g and 4 mg/100 g, respectively). Even when not present at high amounts, the investigated plants can also contribute to the daily intake of thiamine and riboflavin. The rich profile and high contents of bioactive compounds in these WEPs clearly justify their potential use as food ingredients in a healthy and sustainable modern cuisine and in the development of new functional foods.

## 1. Introduction

The Millennium Ecosystem Assessment is a study promoted by the United Nations where more than 1300 researchers from 95 different countries evaluated the consequences of the change in ecosystems for human well-being. This study determined that wild edible plants (WEPs) suffered a decrease in both consumption and collection, due to rural exodus, modernization of lifestyle and industrialization, among other causes [1]. WEPs are plants that grow without the help of humans, suffering undervaluation over years [2]. However, in many developing countries, due to food shortages, these plants have become a fundamental support to meet the daily food needs of many families [3], also providing minerals, antioxidants, antifungal power, and even, for some of them, preventive activity against different health diseases [4,5,6].

Among bioactive compounds found in WEPs, carotenoids are fat-soluble compounds responsible for the color of many vegetables and fruits. They are present in plants, fungi, algae, animals and bacteria. Approximately one thousand two hundred carotenoids were identified, but only six of them (lycopene, α-carotene, lutein, β-carotene, β-cryptoxanthin and zeaxanthin) represent more than 90% in human plasma [7]. Several studies have linked carotenoids with a better response to cataracts, lower risk of cardiovascular diseases, osteoporosis, prevention and treatment of age-related macular degeneration (AMD) and improvement in the control of hypertension [8].

Other interesting compounds found in WEPs are tocols, among which, eight compounds are the most common: tocopherols (α, β, γ and δ) and tocotrienols (α, β, γ and δ). Recent evidences suggest that only α-tocopherol shows vitamin E activity and it still unknown if other tocols are able to prevent vitamin E avitaminosis in humans like α-T [9]. Tocols are commonly found in oils of plant origin [10]. The incorporation of tocopherols into the diet or through deficiency supplements is essential for the proper functioning of the body. Vitamin E, due to its high antioxidant power, was shown to have effect against obesity. Moreover, it helps at improving or preventing health problems such as cataracts and HIV-AIDS, stimulates the immune system and has therapeutic potential against several degenerative diseases [11]. In plants, tocol’s functions range from maintaining the membrane integrity, photo-protection of chloroplasts, to the regulation of the electron transport [11].

The main culinary use of WEPs is in dishes (e.g., soups, salads), where the associated oil ingredient increases the rate of absorption of tocols and carotenoids.

The importance of B1 (thiamine) and B2 (riboflavin) vitamins comes from their essential functions in the human body and problems associated with their deficiency. Thiamine is essential in energy generation, amino acid interconnections and neurological functions. Its deficiency is responsible of a disease called “beriberi” [12]. Riboflavin is essential for the metabolism of lipids, carbohydrates and amino acids and exerts antioxidant protection of cells. Its deficiency leads to skeletal deformities, anemia, ataxia, photophobia or inflammation at the gastrointestinal level, among other symptoms. Its supplementation has been reported to be effective in combating migraines and improving neurological motor disability associated with multiple sclerosis [13].

Within WEPs, the most widespread family is that of Asteraceae. Outside this family, plants, such as *Diplotaxis erucoides* and *Oxalis pes-caprae*, are of importance due to their presence in many countries around the world, especially those of the Mediterranean area. *Diplotaxis* is a therophyte plant belonging to the Brassicaceae family, with a flower with four white and violet petals. It is commonly called “Mediterranean wasabi” for its characteristic spicy flavor. *Oxalis* is a geophyte plant belonging to the Oxalidaceae family, from South Africa, with five-petal yellow flowers and a characteristic acidic flavor. In the past, the importance of these plants was much greater due to their use as ingredients in typical dishes, such as “paella”, as an aromatic plant. In 2022, the Basque Culinary Center published a book called “*Silvestre*” about the botany of these plants, with recipes using wild edible plants [14]. Currently, for both plants, flowers are used as a decoration, while leaves are used in salads.

Considering the importance of these plants and their compounds in human physiology, in this study, the profiles and contents of carotenoids, tocols, thiamine and riboflavin of *Diplotaxis erucoides* and *Oxalis pes-caprae* were investigated. These compounds were analyzed in different botanical parts of the two wild edible plants. Results obtained will contribute to the knowledge of the distribution of these bioactives along the plant and will allow the development of new strategies focused on the valorization of the phytochemical profile of these WEPs.

## 2. Materials and Methods

### 2.1. Plant Material

The investigated *Diplotaxis erucoides* DC. and *Oxalis pes-caprae* L. plants were collected at the Miguel Hernández University, Orihuela campus (38°4′10″ N, 0°59′1″ O, Alicante, Spain) during February 2022. Flowers, leaves and stems of *Oxalis pes-caprae* were investigated; for *Diplotaxis erucoides*, the parts were pods, leaves and stems (Supplementary Figures S1 and S2). Fifty grams of pods of *Diplotaxis erucoides* and fifty grams of flowers of *Oxalis pes-caprae*, two hundred grams of leaves and stems of both plants, chosen randomly, were manually collected. The collection of both plants was carried out, in phenological terms, at the height of flowering. The non-edible parts were discarded (roots) Some aliquots were freeze-dried through a Christ Alpha 2–4 apparatus (B. Braun Biotech International, Melsungen, Germany) for 48 h, to a constant weight, and grounded by means of a refrigerated mill (Taurus Aromatic, Oliana, Spain), mixed and stored at −20 °C until analysis. Some aliquots were immediately stored at –20 °C as a reserve material to be eventually freeze dried and processed as above. The AOAC method was used to determine moisture [15].

### 2.2. Chemicals 

Solvents and other analytical grade reagents, all-trans-β-carotene, thiamine and riboflavin standards were from Sigma (Sigma Aldrich, St. Luis, MO, USA). α-Carotene, 9-*cis*-β-carotene and antheraxanthin, 13-*cis*-β-carotene, neoxanthin, violaxanthin standards were from CaroteNature (Lupsingen, Switzerland). Lutein, zeaxanthin, and β-cryptoxanthin were provided by Extrasynthese (Z.I. Lyon-Nord, Genay, France); α, β, γ and δ-tocopherol standards were from Merck (Darmstadt, Germany); α, β, γ and δ-tocotrienol standards were obtained as in Panfili et al. [16].

### 2.3. Determination of Carotenoids

Carotenoids were extracted by using the saponification and solvent extraction method of Panfili et al. [17] and Fratianni et al. [18] on 0.1 g of freeze dried samples. The residues were suspended in a *n*-hexane:isopropyl alcohol solution (90:10 *v*/*v*) and analyzed through a normal (for xanthophylls) and a reverse phase (for carotenes) HPLC. A Dionex HPLC (Sunnyvale, CA, USA) analytical system, with an Ultimate 3000 pump system, was used. For reverse phase, the mobile phase was methanol:methylterbutylether:water, at a flow rate of 1 mL/min, under a gradient profile as in Mouly et al. [19], by using a 5 µm, C30 YMC (Hampsted, NC, USA) stainless steel column (250 mm × 4.6 mm internal diameter, id). Samples were suspended in methanol/methylterbutylether 50:50 (*v*/*v*). Under normal phase conditions, the mobile phase was *n*-hexane:isopropyl alcohol in multilinear gradient elution from 10% (A) to 20% (B) of isopropyl alcohol in n-hexane as reported elsewhere [18]. A 5 µm Luna column, with a silica stationary phase (250 mm × 4.6 mm id), was used (Phenomenex, Torrance, CA, USA). Data were processed through a Dionex Chromeleon Version 6.6 chromatography system (Sunnyvale, CA, USA). Carotenoids were spectrophotometrically detected at 450 nm, identified and quantified by means of available standard solutions. Vitamin A activity was expressed as Retinol Equivalent (RE) as in [20].

### 2.4. Determination of Tocols

The extraction and determination of tocols were carried out using the method of Panfili et al. [16]. The extraction conditions, the used silica column and the HPLC system were the same reported for carotenoids. The residues were suspended in a n-hexane:isopropyl alcohol solution (99:1 *v*/*v*). A n-hexane:ethyl acetate:acetic acid solution (97.3:1.8:0.9 *v*/*v*/*v*) was used as the mobile phase, at a flow rate of 1.6 mL/min. Tocols were detected through a RF 2000 spectrofluorimeter (Dionex, Sunnyvale, CA, USA), set at an excitation and emission wavelength of 290 nm and 330 nm, respectively, and identified and quantified through known standard solutions. Data were processed by a Dionex Chromeleon Version 6.6 chromatography system (Sunnyvale, CA, USA). Vitamin E activity was expressed as Tocopherol Equivalent (TE), as in Sheppard et al. [21].

### 2.5. Thiamine and Riboflavin Analysis

Thiamine and riboflavin were extracted as in Panfili et al. [22] on 0.4 g of freeze dried samples. Extracts were separated by a Dionex HPLC (Sunnyvale, CA, USA), with an Ultimate 3000 pump system, at a flow rate of 0.8 mL/min, using methanol:NaOAc (40:60 *v*/*v*) as the mobile phase. A 5 μm C18 Luna Phenomenex (Torrance, CA, USA) stainless steel column (250 mm × 4.6 mm i.d.) was used. Fluorimetric detection was performed (RF 2000 spectrofluorimeter; Dionex, Sunnyvale, CA, USA), at an excitation wavelength of 366 nm and an emission wavelength of 453 nm for thiamine, and at an excitation wavelength of 453 nm and an emission wavelength of 580 nm for riboflavin. A Dionex Chromeleon Version 6.6 chromatography system (Sunnyvale, CA, USA) was used to process data. Thiamine and riboflavin were identified and quantified through known available standards.

### 2.6. Statistical Analysis

Data were subjected to one-way analysis of variance (ANOVA) and to Tukey’s multiple range test to compare means. Differences were statistically significant at *p* < 0.05. All statistical analyses were performed using a StatGraphics Plus 5.0 software (Manugistics. Inc., Rockville, MD, USA).

## 3. Results and Discussion

### 3.1. Profile of Carotenoids and Tocols

Ten carotenoids were identified in *Diplotaxis erucoides* and *Oxalis pes-caprae*: lutein, zeaxanthin, violaxanthin, neoxanthin, β-cryptoxanthin, antheraxanthin, α-carotene, 13-*cis*-β-carotene, β-carotene and 9-*cis*-β-carotene (Table 1). 

In *Diplotaxis erucoides*, the main carotenoids were lutein and β-carotene (70% of total carotenoids) in each part of the plant. Leaves of plants from the same family (Brassicaceae), such as *Eruca sativa* L., or from the same genus, such as *Diplotaxis tenuifolia* L., had similar values of lutein, about 11 and 13 mg/100 g f.w. (fresh weight), respectively, and β-carotene (about 3.6 and 4.2 mg/100 g f.w., respectively) [23]. Compared to the leaves (about 24 mg/100 g f.w.), both pods and stems had about five–ten-fold lower carotenoid contents (about 5 and 3 mg/100 g f.w., respectively). 

The carotenoid profile of *Oxalis pes-caprae* was very similar to that of *Diplotaxis*, with the highest values found in leaves. In this case, lutein, β-carotene and β-cryptoxanthin showed the highest contents, reaching 80% of total carotenoids (TC) in leaves. Lutein concentration in leaves was about 4.8 mg/100 g f.w. In plants of the same genus, such as *Oxalis corniculata*, this value was slightly higher (about 11 mg/100 g f.w.), while no zeaxanthin was detected [24]. In flowers and stems, the total carotenoid values were very similar (about 1.5 mg/100 g f.w). In both the investigated plant leaves, quite high amounts of violaxanthin (28% of TC) and neoxanthin (about 15% of TC) were also found. As for β-carotene, plants, such as *Amaranthus spinosus* (about 7 mg/100 g f.w.), or *Vigna gallinacea* A. Rich., *Trilepisium madagascariense* DC. and *Cleome gynandra* L. (about 35, 25 and 29 mg/100 g d.w. dry weight, respectively) had values very similar to *Oxalis pes-caprae* (about 32 mg/100 g d.w.) [25,26]. 

Several green leafy vegetables were reported as good sources of β-carotene and rich sources of lutein [27,28]. Contents on carotenoids may vary due to the different analytical methods used, genotype, weather conditions, maturity stage, location and seasonality [29]. 

Only α (α-Τ), β (β-Τ) and γ (γ-Τ) tocopherols were found in both plants (Table 2). In *Diplotaxis erucoides* α-Τ and γ-T were the most representative. While α-T was mainly present in leaves, the same amounts of γ-T were found in leaves and pods. Values of α-T found in leaves of both *Diplotaxis* and *Oxalis* (about 24 and 49 mg/100 g d.w., respectively) were similar to those of the leaves of *Sonchus asper*, *Sonchus oleraceus*, *Spinacia oleracea* and *Cichorium intybus* (19, 20, 32 and 33 mg/100 g d.w., respectively) [18,22,27]. 

The same tocol profile of *Diplotaxis* was found in *Oxalis pes-caprae*, with α-T values in leaves (about 50 mg/100 g d.w.) higher than those of leaves of *Oxalis acetosella* (18.6 mg/100 g d.w.) [30], while γ-T contents (about 4.0 mg/100 g f.w.) were close to those found in *Sonchus asper*, *Sonchus oleraceus* and *Spinacia oleracea* (about 3.3, 2.7 and 4.7 mg/100 g f.w., respectively) [22,27]. Gamma tocopherol was also found in Brazilian WEPs, such as *Amaranthus spinosus* L. and *Commelina benghalensis*, at 3.3 and 0.7 μg/100 g f.w., respectively [25]. β-Τ and δ-T were not found in *Oxalis pes-caprae*, while δ-T was reported in *Oxalis acetosella* (140 mg/100 g d.w.) [30]. 

### 3.2. Contents of Thiamine and Riboflavin 

Stems of *Diplotaxis erucoides* showed the highest content of thiamine (about 1.5 mg/kg f.w.), followed by leaves, while pods had no contents (Table 3). Similar values were found in the leaves of *Sonchus asper* and *oleraceus* (1.0 mg/kg f.w.) and *Crepis vesicaria* (1.3 mg/kg f.w.) [22]. 

*Oxalis pes-caprae* flowers had the highest thiamine content, followed by leaves, with no amounts in stems. Levels close to those detected in leaves (about 4 mg/kg d.w.), were reported in *Ipomoea aquatic* Forssk. (4.5 mg/kg d.w.), *Achyranthes aspera* L. (1.3 mg/kg d.w.) or *Enhydra fluctuans* Lou. (4.0 mg/kg d.w.) [31]. 

All parts of both plants showed low riboflavin levels. In *Diplotaxis erucoides*, pods had the highest content, followed by leaves and stems. In *Oxalis pes-caprae* the highest amounts were in leaves, about 0.2 mg/Kg f.w., followed by flowers and stems. Both values in leaves of *Diplotaxis* and *Oxalis* were similar to those of *Sonchus asper*, *Sonchus oleraceus* (0.1 mg/kg f.w.) and *Crepis vesicaria* (0.2 mg/kg f.w.) [22,27]. Higher contents were found in *Ipomoea aquatic* Forssk. (7.0 mg/kg d.w.), *Achyranthes aspera* L. (about 4 mg/kg d.w.) and *Enhydra fluctuans* Lour. (about 10 mg/kg d.w.) [31].

### 3.3. Vitamin A and Vitamin E Activity

**Figure 1:**
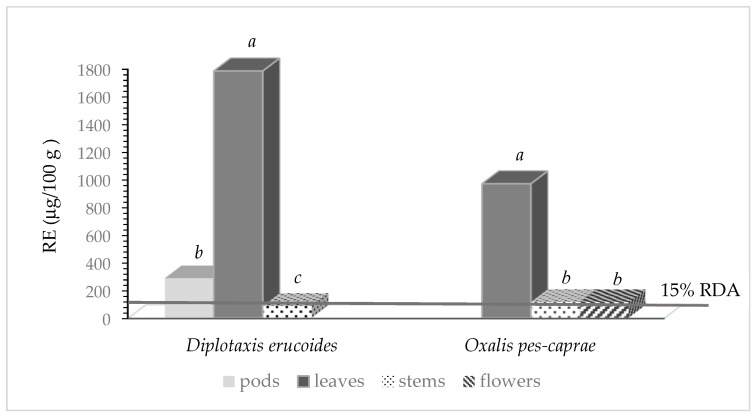
Vitamin A activity (RE) of *Diplotaxis erucoides* and *Oxalis pes-caprae* (μg/100 g). Bars marked with different letters are statistically different at *p* < 0.05.

Figure 1 shows the vitamin A activity of *Diplotaxis erucoides* and *Oxalis pes-caprae*, expressed as Retinol Equivalents (RE). In *Diplotaxis erucoides*, the highest values of RE were reached in leaves, 1783 μg/100 g. Lower values were found in pods and stems (about 290 and 86 μg/100 g, respectively). In the Annex XIII of the Regulation (EU) No 1169/2011, published by the European Union [32], the Recommended Daily Amounts (RDA) for vitamin A, expressed as RE, are 800 μg/day. By ingesting 100 g of fresh *Diplotaxis erucoides* leaves, more than double (223%) of the RDA for vitamin A can be achieved. One hundred grams of pods, instead, covered the 35% of the RDA. Similarly, in *Oxalis pes-caprae*, the highest vitamin A activity was shown in leaves (970 μg/100 g f.w.), so to cover, with 100 g, about 120 % of the RDA for this vitamin. On the contrary, both flowers and stems had lower RE values (about 115 μg/100 g f.w.). The European Union Regulation specifies that any food source that reaches the 15% of the RDA can be declared on the label as a “source of vitamin A” [32]. Therefore, for both *Diplotaxis erucoides* and *Oxalis pes-caprae* it can be stated that 100 g of their leaves are sources of vitamin A.

The found vitamin A levels are far below the Tolerable Upper Intake Levels (ULs) for preformed vitamin A, which are applied only to products from animal sources and supplements whose vitamin A comes entirely from retinol or its ester forms. Nevertheless, there are no upper limits for beta-carotene and other forms of provitamin A supplementation because of the lack of relevance of studies for human risk assessment [33].

**Figure 2:**
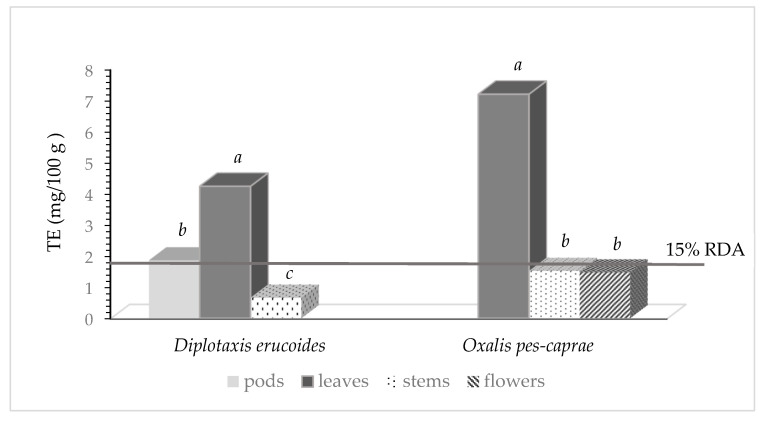
Vitamin E activity (TE) of *Diplotaxis erucoides* and *Oxalis pes-caprae* (mg/100 g). Bars marked with different letters are statistically different at *p* < 0.05.

Figure 2 shows the vitamin E activity of *Diplotaxis erucoides* and *Oxalis pes-caprae*, expressed as Tocopherol Equivalents (TE), as mg per 100 g of fresh weight. In *Diplotaxis erucoides* the highest value of TE was shown in leaves, reaching 4.2 mg/100 g f.w., while it was 1.9 mg/100 g f.w. in pods and 0.7 mg/100 g f.w. in stems. By ingesting 100 g of leaves of *Diplotaxis erucoides*, a consumer can achieve 35% of 12 mg, which is the RDA for vitamin E [32]. Similarly to what was found in *Diplotaxis erucoides*, in *Oxalis pes-caprae*, the highest TE value was reached in leaves (7.2 mg/100 g f.w.), so 60% of the RDA for vitamin E can be covered by 100 g of *Oxalis* leaves. Both flowers and stems had lower values of vitamin E activity (about 1.5 mg/100 g f.w.). The European Union Regulation [32] specifies that any food source that reaches a value greater than 15% of the RDA can be declared on the label as a “source of vitamin E”. Therefore, 100 g of leaves of both *Diplotaxis erucoides* and *Oxalis pes-caprae*, can be stated as sources of vitamin E.

## 4. Conclusions

Results on carotenoids, tocols, thiamine and riboflavin of different parts of *Diplotaxis erucoides* and *Oxalis pes-caprae* show that the profiles and contents of these bioactive compounds are of interest. The highest levels of almost all compounds were mainly found in the leaves. In both plants the major carotenoids were lutein and β-carotene. Among tocols, α-tocopherol was the main compound in all analyzed parts, with the exception of the flowers of *Oxalis*, where γ-tocopherol was predominant. One hundred grams of leaves of *Diplotaxis erucoides* and *Oxalis pes-caprae* provide over the 15% of the Recommended Daily Allowance for vitamin E and vitamin A, so to be considered as a source of these vitamins. These plants can also contribute to the daily intake of thiamine and riboflavin. 

Finding results could be used to improve the nutritional databases and evidence a promising future for WEPs in consumer demand for healthy foods, produced and processed with sustainable methods.

## Figures and Tables

**Table 1:** Content of carotenoids in *Diplotaxis erucoides* and *Oxalis pes-caprae* (mg/100 g f.w., d.w.).

	ANOVA	*p*-Value	Fresh (mg/100 g f.w.)			ANOVA	*p*-Value	Dry (mg/100 g d.w.)		
	*Diplotaxis erucoides*									
			Pods	Leaves	Stems			Pods	Leaves	Stems
Lutein	***	0.0000	2.12 ± 0.03 ^b^	8.23 ± 0.96 ^a^	1.53 ± 0.02 ^b^	***	0.0000	9.38 ± 0.14 ^b^	47.38 ± 5.50 ^a^	8.52 ± 0.13 ^b^
Zeaxanthin	***	0.0000	0.24 ± 0.01 ^b^	0.37 ± 0.00 ^a^	0.11 ± 0.01 ^c^	***	0.0000	1.04 ± 0.06 ^b^	2.14 ± 0.01 ^a^	0.63 ± 0.08 ^c^
Violaxanthin	***	0.0000	0.11 ± 0.01 ^b^	2.28 ± 0.19 ^a^	0.20 ± 0.03 ^b^	***	0.0000	0.50 ± 0.05 ^b^	13.10 ± 1.09 ^a^	1.10 ± 0.15 ^b^
Neoxanthin	***	0.0000	nd	1.07 ± 0.04 ^a^	0.15 ± 0.02 ^b^	***	0.0000	nd	6.14 ± 0.26 ^a^	0.82 ± 0.11 ^b^
β-Cryptoxanthin	ns	0.2879	nd	0.09 ± 0.01 ^a^	0.08 ± 0.01 ^a^	ns	0.1846	nd	0.53 ± 0.09 ^a^	0.43 ± 0.06 ^a^
Antheraxanthin	***	0.0000	0.15 ± 0.00 ^b^	0.38 ± 0.04 ^a^	0.15 ± 0.02 ^b^	***	0.0000	0.67 ± 0.01 ^b^	2.19 ± 0.20 ^a^	0.39 ± 0.05 ^b^
α-Carotene	***	0.0000	0.18 ± 0.02 ^b^	1.00 ± 0.06 ^a^	0.04 ± 0.00 ^c^	***	0.0000	0.79 ± 0.10 ^b^	5.77 ± 0.35 ^a^	0.23 ± 0.01 ^c^
13-*cis*-β-Carotene	***	0.0000	0.05 ± 0.01 ^b^	1.28 ± 0.08 ^a^	nd	***	0.0000	0.24 ± 0.02 ^b^	7.38 ± 0.44 ^a^	nd
β-Carotene	***	0.0000	1.32 ± 0.14 ^b^	9.47 ± 0.57 ^a^	0.39 ± 0.01 ^c^	***	0.0000	5.82 ± 0.63 ^b^	54.48 ± 3.27 ^a^	2.18 ± 0.01 ^b^
9-*cis*-β-Carotene	***	0.0000	0.57 ± 0.07 ^a^	0.09 ± 0.01 ^b^	0.13 ± 0.01 ^b^	***	0.0000	2.54 ± 0.32 ^a^	0.51 ± 0.03 ^b^	0.72 ± 0.01 ^b^
Totals	***	0.0000	4.75 ± 0.28 ^b^	24.26 ± 1.85 ^a^	2.70 ± 0.05 ^b^	***	0.0000	20.97 ± 1.25 ^b^	139.63 ± 10.62 ^a^	15.04 ± 0.33 ^b^
	*Oxalis pes-caprae*									
			Flowers	Leaves	Stems			Flowers	Leaves	Stems
Lutein	***	0.0000	0.10 ± 0.02 ^b^	4.76 ± 0.29 ^a^	0.38 ± 0.05 ^b^	***	0.0000	0.72 ± 0.12 ^c^	34.20 ± 2.07 ^a^	4.25 ± 0.59 ^b^
Zeaxanthin	**	0.0085	0.17 ± 0.01 ^a^	0.15 ± 0.03 ^a^	0.10 ± 0.01 ^b^	ns	0.6089	1.21 ± 0.08 ^a^	1.12± 0.21 ^a^	1.11 ± 0.01 ^a^
Violaxanthin			nd	nd	0.04 ± 0.01			nd	nd	0.49 ± 0.03
Neoxanthin			nd	nd	nd			nd	nd	nd
β-Cryptoxanthin	***	0.0001	0.16 ± 0.03 ^b^	1.35 ± 0.24 ^a^	0.32 ± 0.05 ^b^	***	0.0000	1.11 ± 0.23 ^c^	9.74 ± 1.74 ^a^	3.53 ± 0.06 ^b^
Antheraxanthin	***	0.0001	nd	0.43 ± 0.06 ^a^	0.02 ± 0.01 ^b^	***	0.0004	nd	3.12 ± 0.46 ^a^	0.24 ± 0.02 ^b^
α-Carotene	***	0.0004	0.05 ± 0.01 ^b^	0.34 ± 0.08 ^a^	0.05 ± 0.01 ^b^	***	0.0006	0.37 ± 0.01 ^b^	2.45 ± 0.61 ^a^	0.58 ± 0.01 ^b^
13-*cis*- β-Carotene	***	0.0000	0.05 ± 0.01 ^a^	0.01 ± 0.01 ^b^	0.01 ± 0.01 ^b^	***	0.0000	0.34 ± 0.01 ^a^	0.10 ± 0.01 ^c^	0.13 ± 0.01 ^b^
β-Carotene	***	0.0005	0.49 ± 0.01 ^b^	4.50 ± 1.19 ^a^	0.44 ± 0.05 ^b^	***	0.0006	3.48 ± 0.03 ^b^	32.23 ± 8.55 ^a^	4.90 ± 0.56 ^b^
9-*cis*-β-Carotene	**	0.0014	0.12 ± 0.01 ^b^	0.93 ± 0.29 ^a^	0.11 ± 0.01 ^b^	***	0.0000	0.88 ± 0.01 ^c^	6.69 ± 0.22 ^a^	1.26 ± 0.08 ^b^
Totals	***	0.0000	1.47 ± 0.07 ^b^	12.50 ± 1.71 ^a^	1.49 ± 0.11 ^b^	***	0.0000	8.11 ± 0.47 ^c^	89.73 ± 10.23 ^a^	16.49 ± 1.21 ^b^

Data are shown as mean of 3 replicates ± standard deviation. **, *** significant at *p* < 0.01 and 0.001, respectively. nd (not detected). ns (not significant at *p* < 0.05). Values followed by the same letter, within the same row, were not significantly different.

**Table 2:** Tocol content in *Diplotaxis erucoides* and *Oxalis pes-caprae* (mg/100 g f.w., d.w.).

	ANOVA	*p*-Value	Fresh (mg/100 g f.w.)			ANOVA	*p*-Value	Dry (mg/100 g d.w.)		
	*Diplotaxis erucoides*									
			Pods	Leaves	Stems			Pods	Leaves	Stems
α-Tocopherol	***	0.0000	1.81 ± 0.13 ^b^	4.13 ± 0.44 ^a^	0.65 ± 0.06 ^c^	***	0.0000	7.97 ± 0.57 ^b^	23.79 ± 2.53 ^a^	3.62 ± 0.33 ^c^
β-Tocopherol	***	0.0000	nd	0.10 ± 0.01 ^a^	0.01 ± 0.01 ^b^	***	0.0000	nd	0.60 ± 0.02 ^a^	0.06 ± 0.01 ^b^
γ-Tocopherol	***	0.0000	0.56 ± 0.03 ^a^	0.50 ± 0.07 ^a^	0.04 ± 0.01 ^b^	***	0.0000	2.47 ± 0.12 ^a^	2.89 ± 0.40 ^a^	0.21 ± 0.02 ^b^
Totals	***	0.0000	2.37 ± 0.16 ^b^	4.74 ± 0.51 ^a^	0.70 ± 0.05 ^c^	***	0.0000	10.44 ± 0.70 ^b^	27.28 ± 2.94 ^a^	3.89 ± 0.25 ^c^
	*Oxalis pes-caprae*									
			Flowers	Leaves	Stems			Flowers	Leaves	Stems
α-Tocopherol	***	0.0000	1.17 ± 0.13 ^b^	6.81 ± 0.44 ^a^	1.41 ± 0.01 ^b^	***	0.0000	8.25 ± 0.92 ^c^	48.89 ± 3.16 ^a^	15.55 ± 0.02 ^b^
β-Tocopherol			nd	nd	nd			nd	nd	nd
γ-Tocopherol	***	0.0003	3.05 ± 0.56 ^a^	3.91 ± 0.28 ^a^	1.11 ± 0.16 ^b^	***	0.0002	21.59 ± 2.96 ^b^	28.09 ± 1.99 ^a^	12.23 ± 1.81 ^c^
Totals	***	0.0000	4.22 ± 0.33 ^b^	10.72 ± 0.11 ^a^	2.51 ± 0.16 ^c^	***	0.0000	29.84 ± 1.89 ^b^	76.98 ± 1.17 ^a^	27.78 ± 1.83 ^b^

Data are shown as mean of 3 replicates ± standard deviation. *** significant at *p* < 0.001, respectively. nd (not detected). Values followed by the same letter, within the same row, were not significantly different.

**Table 3:** Contents of thiamine and riboflavin in *Diplotaxis erucoides* and *Oxalis pes-caprae* (mg/kg f.w., d.w.).

	ANOVA	*p*-Value	Fresh (mg/kg f.w.)			ANOVA	*p*-Value	Dry (mg/kg d.w.)		
	*Diplotaxis erucoides*									
			Pods	Leaves	Stems			Pods	Leaves	Stems
Thiamine	***	0.0000	nd	0.64 ± 0.06 ^b^	1.46 ± 0.04 ^a^	***	0.0000	nd	3.72 ± 0.15 ^b^	8.13 ± 0.21 ^a^
Riboflavin	***	0.0000	0.23 ± 0.02 ^a^	0.14 ± 0.01 ^b^	0.03 ± 0.01 ^c^	***	0.0000	1.02 ± 0.10 ^a^	0.83 ± 0.05 ^b^	0.18 ± 0.06 ^c^
	*Oxalis pes-caprae*									
			Flowers	Leaves	Stems			Flowers	Leaves	Stems
Thiamine	***	0.0000	1.17 ± 0.05 ^a^	0.33 ± 0.02 ^b^	nd	***	0.0000	8.25 ± 0.33 ^a^	2.36 ± 0.16 ^b^	nd
Riboflavin	***	0.0000	0.10 ± 0.01 ^b^	0.16 ± 0.01 ^a^	0.02 ± 0.01 ^c^	***	0.0000	0.69 ± 0.01 ^b^	1.14 ± 0.06 ^a^	0.26 ± 0.01 ^c^

Data are shown as mean of 3 replicates ± standard deviation. *** significant at *p* < 0.001, respectively. nd (not detected). Values followed by the same letter, within the same row, were not significantly different.

## Data Availability

Data are contained within the article.

## Supplementary Materials

The following supporting information can be downloaded at: https://www.mdpi.com/article/10.3390/nu16142293/s1. Figure S1. Parts of *Oxalis pes-caprae* L. Source: www.antropocene.it; Figure S2. Parts of *Diplotaxis erucoides* DC. Source: Flickr.
