# Supplementary material for: Diplotaxis erucoides and Oxalis pes-caprae: Two Wild Edible Plants as a New and Valuable Source of Carotenoids, Tocols and B1 and B2 Vitamins
Source: Nutrients. 2024 Jul 17;16(14):2293. doi: 10.3390/nu16142293 (PMC11279960; doi:10.3390/nu16142293)
Supplement: Supplementary file 1 [file nutrients-16-02293-s001.zip › nutrients-3103310-supplementary.pdf]

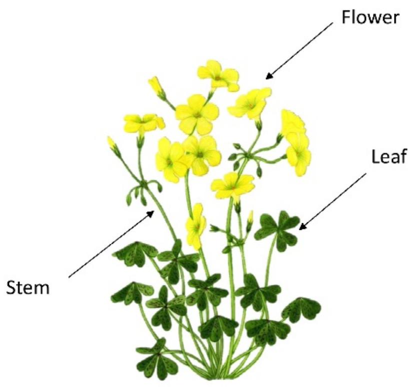

Figure S1. Parts of *Oxalis pes-caprae* L. Source: www.antropocene.it

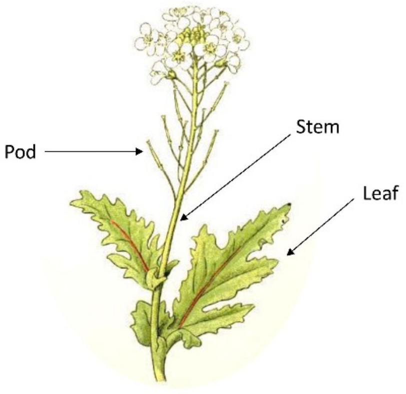

Figure S2. Parts of *Diplotaxis erucoides* DC. Source: Flickr.
